# A Pressure Control Method for Emulsion Pump Station Based on Elman Neural Network

**DOI:** 10.1155/2015/684096

**Published:** 2015-03-09

**Authors:** Chao Tan, Nan Qi, Xin Zhou, Xinhua Liu, Xingang Yao, Zhongbin Wang, Lei Si

**Affiliations:** ^1^School of Mechatronic Engineering, China University of Mining & Technology, Xuzhou 221116, China; ^2^Xuyi Mine Equipment and Materials R&D Center, China University of Mining & Technology, Huai'an 211700, China

## Abstract

In order to realize pressure control of emulsion pump station which is key equipment of coal mine in the safety production, the control requirements were analyzed and a pressure control method based on Elman neural network was proposed. The key techniques such as system framework, pressure prediction model, pressure control model, and the flowchart of proposed approach were presented. Finally, a simulation example was carried out and comparison results indicated that the proposed approach was feasible and efficient and outperformed others.

## 1. Introduction

As key equipment to ensure coal mine safety production and achieve high efficiency, emulsion pump station provides high pressure emulsion for hydraulic support and hydraulic jack. The control system performances of quick response and steady tracking have direct influence on moving speed and bracing force [1]. Therefore, reasonable control method of emulsion pump station is essential to the whole coal mine.

Though tremendous progress has been made, great fluctuations of hydraulic system pressure still exist in actual use [2], which brings safety dangers to the whole coal mine. The existing system pressure control of emulsion pump station is a constant-pressure control method based on PID pressure compensation, the principle of which is to set expected pressure values for different working systems. When measured pressure value is too low or too high, which means the value is beyond error control, they change control pump unit motor frequency so that it increases or reduces pressure output to make the system pressure return into the appropriate operating range [3–5]. Essentially this method is a type of pressure compensation based on pressure itself, which cannot meet the demand of control in real time. On one hand, the emulsion pressure should come to the point that hydraulic support can achieve right motions in coal mining [6]. On the other hand, unnecessary emulsion pressure can bring resource waste and great fluctuations in the system [7]. That is, reasonable pressure emulsion is the key issue. At the same time, as a typical local recurrent network, Elman neural network is widely used.

Bearing the above observations in mind, Elman neural network is used for emulsion pump station to control pressure. The effect of hydraulic supports motions on system pressure is analyzed based on the field data. Combining the motions time and pressure set, we can obtain reasonable expected pressure. The model can not only follow the load, but also save energy.

The paper is structured as follows: some related works are outlined based on literature in Section 2. The factors to impact emulsion pump station pressure change are analyzed in Section 3. The framework and key technologies are proposed, and the flowchart of proposed approach is designed in Section 4. A simulation example and some comparisons are put forward to validate the proposed approach in Section 5. Our conclusions are summarized in Section 6.

## 2. Literature Review

In this section, we try to list and summarize some recent papers which are relevant to the control methods of pressure system. Generally, scholars make efforts to control pressure from two aspects; one is to improve the performance of system to follow the load [8–10] and the other is to propose new algorithms [11–13] to realize pressure control.

### 2.1. Pump Station Control Methods

Nowadays, pressure control methods of hydraulic pump station have maturely developed. Because of difficult data obtaining and other reasons, pressure control of emulsion pump station needs to be improved. To save the energy, a logical method and electric design were provided to realize automatic control [14, 15]. In [16], a fusion approach of fuzzy control and PID was applied in emulsion pump station which got perfect dynamic performance and no state error. Giving hardware structure and software design, Xu et al. [17] researched frequency-conversion speed-regulation system of emulsion pump station. In the respect of system improving, Yang [18] analyzed the state of unloading valve and recovery pressure to ensure the stability of emulsion pump station. With the same working principle, emulsion pump station transfers power with emulsion, while other pump stations use hydraulic oil. A remote pressure control system was designed for capillary pressure in [19], and the result brought possibility to the creation of superior automatic microinjection controllers. Umrao and Chaturvedi [20] studied a novel fuzzy control approach for load frequency control, which avoided large number of rules. Yang et al. [21] also got good robustness from applying adaptive fuzzy control technology to pressure control of a pressurizer and the results showed that the controller was effective for the kind of systems with nonlinear, complex, and imprecise mathematical model. We can see that introduction of intelligent algorithm makes pump station pressure control have broader prospects.

### 2.2. Neural Network Control

As artificial neural network has the function of simulating human thinking and incomparable superiority in terms of establishing nonlinear and experiential knowledge simulation models, it is widely used in many fields, such as time series problems [22], prediction of field emission [23], prediction of mechanic parts performance [24], hydraulic winch control [25], and PMLSM position tracking control [26]. Nowadays, recurrent neural network is one of the study hotspots in the field of control. First proposed by Elman in 1990 [27], Elman neural network was the typical local recursion delay feedback neural network [28].

### 2.3. Discussion

Although many approaches for pressure control systems have been presented in the above papers, they have a common disadvantage summarized as follows. With the above approaches, the pressure fluctuations are too large to realize continuous right motions of hydraulic support. Moreover, large pressure fluctuations would impact the whole hydraulic system, especially the hydraulic valve and pipe, which accelerate abrasion of key components and reduce life of the equipment.

In order to reduce the pressure fluctuations, a pressure control method for emulsion pump station based on Elman neural network is proposed and a simulation example is provided to verify the effect of proposed method.

## 3. Emulsion Pump Station and Hydraulic Support

Emulsion pump station pressure is influenced by both hydraulic support motions and its pressure loss. The pressure loss contains pipeline pressure loss and valve pressure loss. At different oil pressures, different pressure losses occur. But we see the pressure loss as a part of hydraulic support motions influence in this paper. That is, pump station pressure is totally influenced by support states. And support states influence varies with the variation of the oil pressure.

In order to make it convenient for the following, we encode hydraulic states in Table 1.

## 4. The Proposed Pressure Control Approach

This section tries to present a new method aiming at providing stable emulsion pressure. The section has three main parts and can be elaborated through the following subsections.

### 4.1. The Framework of Proposed Approach

The construction process of control system is shown as follows.

**Figure 1:**
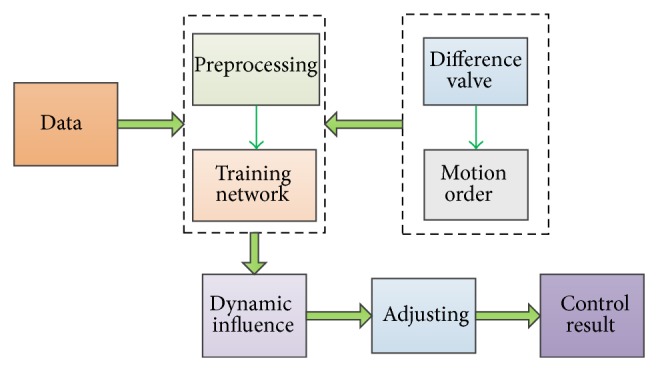
The framework of proposed approach.

1. We get groups of data from industrial field. Because of large quantity and noise impact, data preprocessing is done before applying to experiments which means dropping data beyond range.
2. After Elman neural network programming, 5040 groups of data are chosen as sampling to train the network. The relationship between oil pressure difference and motion order is simulated, and dynamic motion difference value matrix is obtained.
3. Giving operating conditions, we know motion order of hydraulic supports. When oil pressure is not equal to set pressure, the difference is returned to the network. According to the network, dynamic motion influence valve is obtained. Regulating the frequency of frequency converter to reduce difference valve, we can get steady oil pressure.  Figure 1 shows the framework of proposed approach.

### 4.2. Prediction of Emulsion Pressure

**Figure 2:**
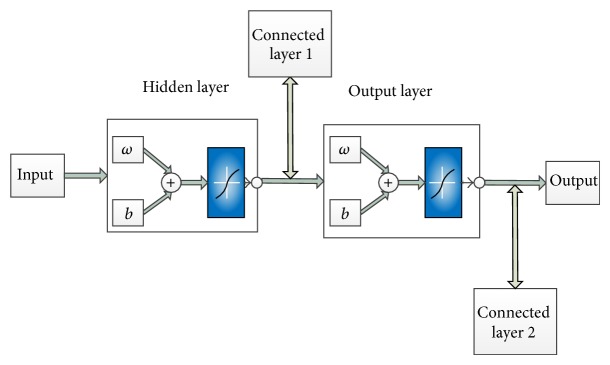
Principle of Elman neural network.

Neural network is an ideal type of nonlinear approximator. The important components are input layer, hidden layer, connected layer, and output layer. Compared with BP neural network, connected layer is added for partial feedback in Elman neural network [29]. Transfer function of it is linear, and to remember the state of past time series delay units are included. Having both memory unit and input of network as input of hidden layer, we can see dynamic memory function [30] in Elman neural network. Transfer function of connected and output layer is linear, while that of hidden layer is nonlinear, such as hyperbolic S nonlinear function, step function, and prelinear function. Because of hidden layer receiving the data from input and memory data in connected layer, the same input can bring different output. The principle of Elman neural network with the characteristic of multi-input and single-output is shown in Figure 2.

Due to linear network, finite and discontinuous functions can be expressed in Elman neural network. For multi-input and single-output networks, we set *X*_*c*_ as input of connected layer, *X*_*h*_ as input of hidden layer, *Y*_*h*_ as output of hidden layer, and *Y* as exterior output of motion series *U*; then we get the formula of hidden layer input:

(1) $$\begin{array}{c} X_{h}\left(k\right)=f\left(W^{1}X_{c1}\left(k\right)+W^{2}U\left(k-1\right)+W^{4}X_{c2}\left(k\right)\right), \end{array}$$

where *X*_*c*1_(*k*) is output of connected layer 1,

(2) $$\begin{array}{c} X_{c1}\left(k\right)=\alpha_{y}X_{c1}\left(k-1\right)+Y_{h}\left(k-1\right), \end{array}$$

and *X*_*c*2_(*k*) is output of connected layer 2. Consider

(3) $$\begin{array}{c} X_{c2}\left(k\right)=\gamma X_{c2}\left(k-1\right)+Y_{h}\left(k-1\right),\\ Y\left(k\right)=g\left(W_{3}Y_{h}\left(k\right)\right). \end{array}$$

In the formulas, *W* ^1^ is connected weight matrix of connected unit 1 and hidden layer, *W* ^2^ of input layer and hidden layer, *W* ^3^ of output layer and hidden layer, and *W* ^4^ of connecting unit 2 and hidden layer. *α*_*y*_ is the feedback parameter of connected unit 1, while *γ* is that of connected unit 2. *f*(·) is the function of activation. Here we set *f*(·) as Sigmoid function [31]:

(4) $$\begin{array}{c} f\left(x\right)=\frac{1}{1+e^{-x}}. \end{array}$$

If *y*_*g*_(*k*) is the output after the *k* steps, error function can be expressed in Elman neural network:

(5) $$\begin{array}{c} E\left(x\right)=\frac{1}{2}{\left(y_{g}\left(k\right)-y\left(k\right)\right)}^{T}\left(y_{g}\left(k\right)-y\left(k\right)\right). \end{array}$$

With the application of error function to derivate *W*_1_, *W*_2_, *W*_3_, and *W*_4_, we know the learning expression by using the gradient descent algorithm:

(6) ΔWjl1=η1∑i=1mδi0Wij3∂Xc1jk∂Wjl1j=1,2⋯n;  l=1,2⋯m,ΔWjq2=η2δjhUqk−1, j=1,2⋯n;  q=1,2⋯r,ΔWij3=η3δi0Yhjk, j=1,2⋯m;  j=1,2⋯n,ΔWjl4=η4∑i=1mδi0Wij3∂Xc2jk∂Wjl4j=1,2⋯n;  l=1,2⋯m.

In the formulas, *η*_1_ is learning step-size of *W* ^1^, *η*_2_ of *W* ^2^, *η*_3_ of *W* ^3^, and *η*_4_ of *W* ^4^, and *δ*_*i*_^0^, *δ*_*j*_^*h*^, ∂*X*_*c*2*j*_(*k*)/∂*W*_*js*_^4^, and ∂*X*_*c*1*j*_(*k*)/∂*W*_*jl*_^1^ are relevant functions. We can get the following expressions:

(7) $$\begin{array}{c} \delta_{i}^{0}=\left(Y_{g,i}\left(k\right)-Y_{i}\left(k\right)g_{i}^{\prime }\left(\cdot \right)\right),\\ \delta_{i}^{h}=\overset{m}{\underset{i=1}{\sum }}\left(\delta_{i}^{0}W_{ij}^{3}\right)f_{j}^{\prime }\left(\cdot \right),\\ \frac{\partial X_{c1j}\left(k\right)}{\partial W_{jl}^{1}}=f_{j}^{\prime }\left(\cdot \right)X_{c1j}\left(k-1\right)+\alpha \frac{\partial X_{c1j}\left(k-1\right)}{\partial W_{jl}^{1}},\\ \frac{\partial X_{c2j}\left(k\right)}{\partial W_{jl}^{4}}=f_{j}^{\prime }\left(\cdot \right)X_{c2j}\left(k-1\right)+\alpha \frac{\partial X_{c2j}\left(k-1\right)}{\partial W_{jl}^{4}}. \end{array}$$

To avoid the problem of local optimal solution coming with local regression, comparison of predictive results is made between training samples and samples without training. If the predictive results are in the range of error threshold, they can be accepted. If not, they must be retrained until meeting the condition.

In the process of prediction, learning objects are defined as the nearest sample. In the set cycle, we update the network to realize the real-time control. The length of training samples is determined by control accuracy and predictive length in the system.

### 4.3. Control of Emulsion Pressure

**Figure 3:**
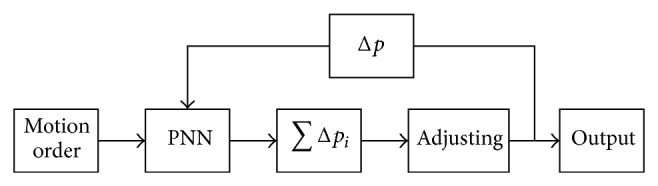
Dynamic compensation of feeding pressure.

Considering motion order of hydraulic support, combining feedback difference of pressure sensor, we get dynamic compensation of feeding pressure. The process is shown in Figure 3.

In Figure 3, pressure sensor is located to detect oil pressure. And Δ*p* is difference value of detected value and expected output. In this paper, output pressure could be stabilized around 28 MPa which is our controlling aim. ∑*p*_*i*_ means sum influence value of hydraulic support motions in the next moment. And then we can adjust corresponding frequency to get the output.

According to regular hydraulic support motion, motion order is known, that is, retracting top coal-wall support, lowering the column, hauling support, raising the column, extending top coal-wall support, and then propelling and pushing.

### 4.4. The Flowchart of Proposed Approach

**Figure 4:**
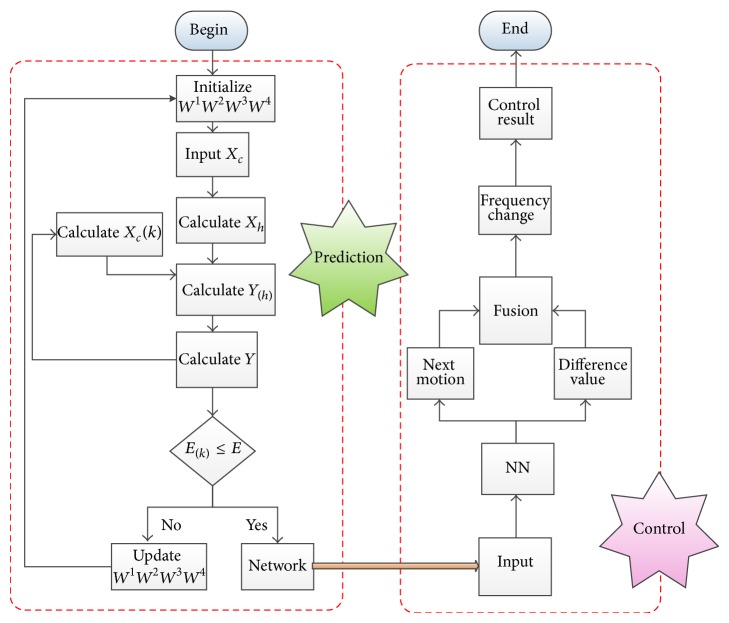
The flowchart of proposed approach.

According to the above description about the approach based on Elman neural network, the proposed approach can be coded easily on the computer, and the flowchart can be summarized as shown in Figure 4.

## 5. A Simulation Example and Industrial Experiment

In this section, an engineering application of emulsion pump station pressure in a coal mine was put forward as a simulation example to verify the feasibility and effectiveness of proposed approach.

We chose six hydraulic supports as research objects. When hydraulic supports were running, the pressure of emulsion pump was detected. According to state coding of Table 1, we made statistics of motions and pressure. As the input layer, 5040 groups of data, gotten from the statistics, were shown in Table 2.

About the coding, the first figure meant the state of the first hydraulic support. Likewise, the second figure meant the state of hydraulic support 2.

Input of the network was motion codes and their pressure differences, and output was every motion difference value in the condition. That is, we could get continuous updated motion difference valve. Through six hidden layer nodes, the network was well trained and the error was controlled in 5%.

**Figure 5:**
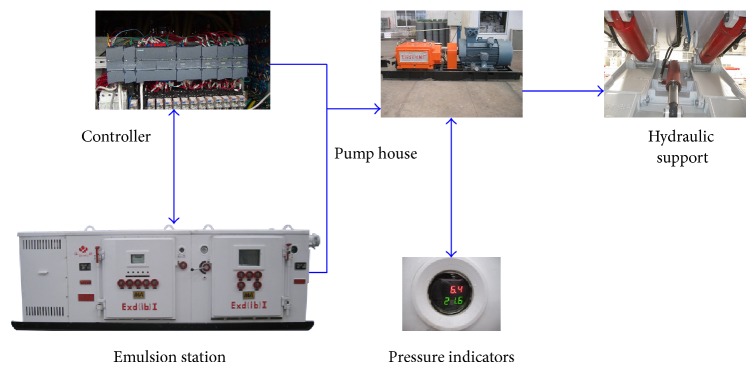
The schematic diagram of control method.

Emulsion pump pressure was influenced by both hydraulic support pressure and its motions. The proposed method was applied to emulsion pump control. The schematic diagram of control method was shown in Figure 5 and control results were shown in Figures 7 and 8.

And industrial experiment was described in detail as follows.

**Step 1 .**

Open emulsion pump station system and hydraulic system, and adjust emulsion pump station and hydraulic supports to condition of state. All of the system pipelines were normal, and no loss existed.

**Step 2 .**

Operations were done to the six hydraulic supports in five minutes, so actions were done in turns. The order of actions was as described in Table 1.

**Step 3 .**

After six actions, the six hydraulic supports would do the following motion combinations, which were 12, 13, 14, 15, 16, 23, 24, 25, 26, 34, 35, 36, 45, 46, 56, 123, 234, 345, 456, and so on.

**Step 4 .**

Statistical analysis was done to experimental data in five minutes. Sampling period was 1 second.

**Figure 6:**
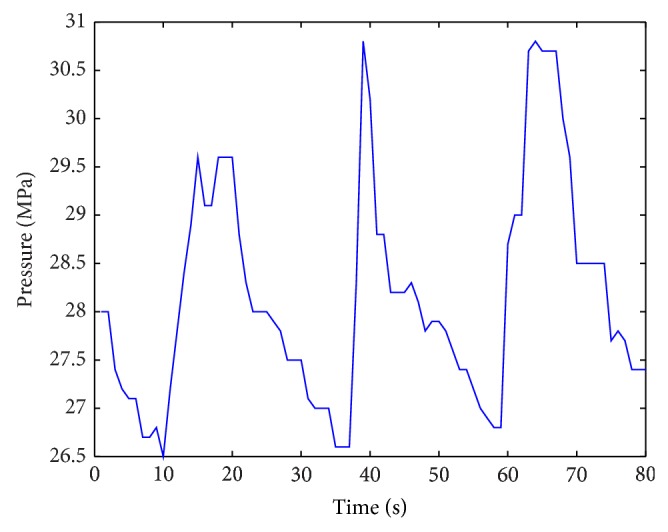
Control result based on hydraulic support motions.

**Figure 7:**
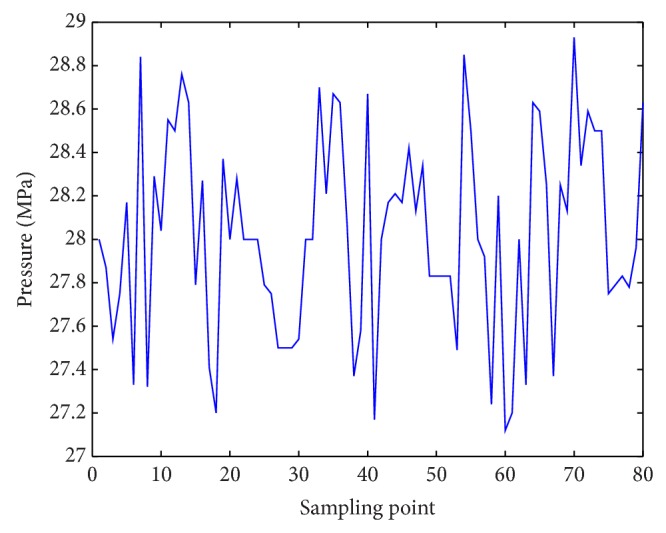
Stable result based on hydraulic support motions.

In the controlling initial period, we got result in Figure 6. For a substantial time, the system became stable in Figure 7.

Analyzing control result, this controller could provide reasonable pressure to hydraulic motions. The proposed control method decreased pressure range which avoided frequent speed changing converter.

**Figure 8:**
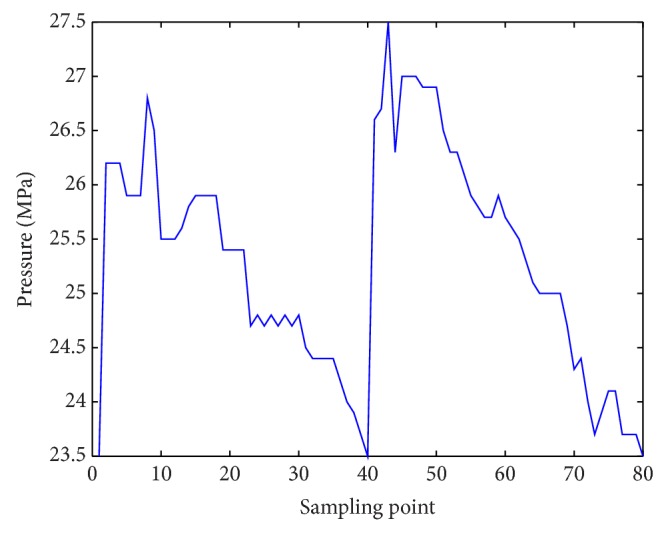
Control result of PID controller.

**Figure 9:**
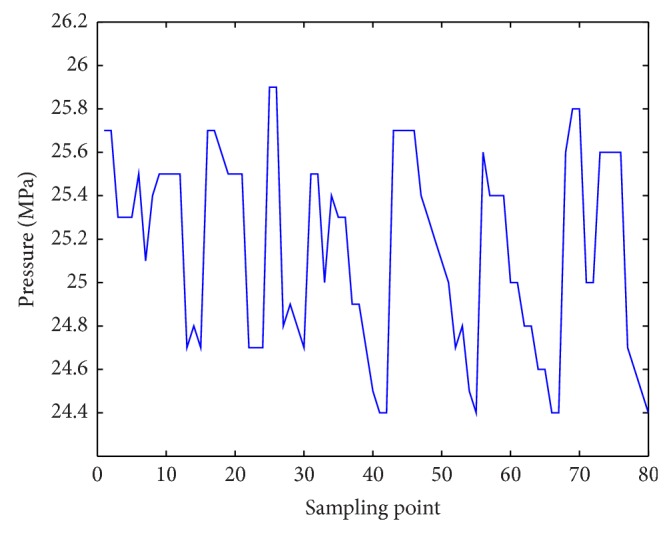
Control result of fuzzy PID controller.

Figures 8 and 9 showed control results of PID and fuzzy PID controller [32]. Obviously, the proposed controller had better dynamic tracking property. Max fluctuation in the process of liquid turned smaller which reduced pipe wearing. Smaller control error was found in prediction control method. In fact, as control methods based on PID ignored pressure loss in pipeline, control error would be larger along with longer pipeline. Control system specifications of three methods were shown in Table 3.

Direct measurement of response time was hard to do. Through calculating relative coefficient of experimental data and goal valve from the following equation, we got relative coefficients in Table 3. In the equation, *r* means relative coefficient. *x*_*i*_ and *y*_*i*_ mean *n*th of two sets of data:

(8) $$\begin{array}{c} r=\frac{\sum_{i=1}^{n}\left(x_{i}-\bar{x}\right)\left(y_{i}-\bar{y}\right)}{\sqrt{\sum_{i=1}^{n}{\left(x_{i}-\bar{x}\right)}^{2}\cdot \sum_{i=1}^{n}{\left(y_{i}-\bar{y}\right)}^{2}}}. \end{array}$$

If goal valve of last second was shown in this second, we calculated that the relative coefficient was 0.23. While relative coefficient of prediction control method was 0.63, that was bigger than 0.23. Obviously response time was less than 1 second.

## 6. Conclusions and Future Work

This paper proposed a control method for emulsion pressure based on Elman neural network considering hydraulic support motions. In order to verify the feasibility and superiority, the proposed approach was applied to an engineering problem of emulsion pressure control. The results of comparison simulations showed that the proposed approach could provide more reasonable pressure than existing PID controller. At the same time, the proposed method could help to save energy and make a contribution to protecting the environment. The control performance of proposed model demonstrated that this method could be extended to process other types of other pressure control.

In further studies, the authors plan to investigate control system considering more intelligent algorithms.

## Figures and Tables

**Table 1:** Coding table of hydraulic states.

States	Code
No motions	0
Retracting top coal-wall support	1
Lowering the column	2
Hauling support	3
Raising the column	4
Extending top coal-wall support	5
Propelling and pushing	6

**Table 2:** Part of input data.

Coding	D-valve (MPa)	Δ*p*_1_	Δ*p*_2_	Δ*p*_3_	Δ*p*_4_	Δ*p*_5_	Δ*p*_6_
000001	+0.7	+0.7	0	0	0	0	0
000002	+1.5	0	+1.5	0	0	0	0
000003	+1.2	0	0	+1.2	0	0	0
000004	−2	0	0	0	−2	0	0
000005	+0.7	0	0	0	0	+0.7	0
000006	−1	0	0	0	0	0	−1
000011	+1.5	+0.75	0	0	0	0	0
000012	+2	+0.6	+1.4	0	0	0	0
000013	+2	+0.75	0	+1.25	0	0	0
⋮	⋮	⋮	⋮		⋮	⋮	
555555	+4.0	0	0	0	0	+0.65	0
666666	−5.7	0	0	0	0	0	−0.95

**Table 3:** Control system specifications of three methods.

Parameter	PID control	Fuzzy PID	Prediction control
Range/MPa	23.5~27.5	24.4~25.9	27.12~28.93
Mean value/MPa	25.295	25.204	28.026
Variance/MPa	1.056	0.448	0.248
Max error/MPa	4.5	3.6	0.93
Relative coefficient	0.02	−0.07	0.63
